# Prevalence of gestational diabetes mellitus in Germany: Temporal trend and differences by regional socioeconomic deprivation

**DOI:** 10.25646/12086

**Published:** 2024-05-15

**Authors:** Lukas Reitzle, Christin Heidemann, Laura Krause, Jens Hoebel, Christa Scheidt-Nave

**Affiliations:** Robert Koch Institute, Berlin, Department of Epidemiology and Health Monitoring

**Keywords:** Pregnancy, Gestational diabetes, Surveillance, Social deprivation, Germany

## Abstract

**Background:**

Gestational diabetes mellitus (GDM) increases the risk for adverse pregnancy outcomes. In 2012, a general screening for GDM was introduced in Germany.

**Methods:**

The analysis is based on data from the external inpatient quality assurance for obstetrics from the years 2013 to 2021. Women with pregestational diabetes were excluded. GDM was defined either by documentation in the maternity record or by ICD diagnosis O24.4 during hospitalisation. We reported the prevalence stratified by year, maternal age and regional socioeconomic deprivation.

**Results:**

The age-standardized prevalence of GDM continuously rose from 4.7 % in 2013 to 8.5 % in 2021. The increase was observed in all age groups. In 2021, this corresponded to 63,563 women with GDM. The prevalence was higher in highly deprived regions than in low deprived regions.

**Conclusion:**

A steady increase in GDM prevalence and evidence of health inequalities emphasise the need for primary prevention strategies for GDM.

## 1. Introduction

Diabetes mellitus during pregnancy is one of the most frequent risk factors for complications during pregnancy and birth. A distinction is made between diabetes type 1 or type 2 existing already before pregnancy (pregestational diabetes) and a dysregulation of the blood glucose metabolism that occurs for the first time during pregnancy – also known as gestational diabetes mellitus (GDM) [1]. Both in Germany and worldwide, the prevalence of GDM has increased in recent years [2, 3]. Important potentially preventable risk factors for incident GDM are obesity, significant weight gain during pregnancy and physical inactivity. Obesity and physical inactivity [4, 5], as well as GDM itself [6], are more frequent in socioeconomically disadvantaged groups.

During pregnancy GDM can lead to increased fetal growth with in consequence higher birth weight of the newborn, which increases the risk of birth injuries. Furthermore, GDM increases the risk of preterm birth [7, 8]. The risk of complications can be significantly reduced by treating GDM [1]. In 2012, a general, two-stage screening for GDM was introduced [9] in order to inform and treat pregnant women with GDM on the basis of guideline recommendations. Although GDM usually disappears after pregnancy, the mother’s risk of developing type 2 diabetes later in life is significantly increased [10]. The affected children may have an increased risk of developing obesity later on [11].

The National Diabetes Surveillance at the Robert Koch Institute monitors the frequency of diabetes, its risk factors and consequences using 40 indicators and indicator groups [12]. The surveillance assesses the prevalence of GDM, the screening participation and pregnancy complications. This present study analyses the development of the prevalence of GDM in Germany over time, taking socioeconomic differences into account.

## 2. Indicator

Data from quality assurance procedures pursuant to Section 136 of the German Social Code (Sozialgesetzbuch, SGB) V of the Federal Joint Committee (Gemeinsamer Bundesausschuss, G-BA) were used for this study. The quality assurance system for obstetrics (perinatal medicine) contains information on the pregnancy based on the maternity record and on the hospital stay during birth [13]. As part of secondary data use, data can be requested from the Institute for Quality Assurance and Transparency in Health Care (IQTIG, Institut für Qualitätssicherung und Transparenz im Gesundheitswesen). For analysis of socioeconomic differences, we used the German Index of Socioeconomic Deprivation (GISD) Release 2022 v0.2, which measures regional socioeconomic deprivation at the level of municipalities and collective municipalities [14, 15]. The GISD was linked with information on GDM using the first four digits of the postal code of the place of residence.

In the analysis, we included data on all hospital births from 2013 to 2021. As in previous analyses, we excluded mothers with pregestational diabetes documented in the first antenatal visit in catalogue A of the maternity record (0.9 % – 1.0 % per year) [3, 16]. GDM was considered present if documented in the maternity record in catalogue B ‘Special findings during pregnancy’ or as International Statistical Classification of Diseases and Related Health Problems, 10th revision (ICD-10) diagnosis O24.4 during hospitalisation.

The data was were provided in aggregated format by the IQTIG stratified by reporting year and maternal age groups (< 20, 20 – 24, 25 – 29, 30 – 34, 35 – 39, 40 – 44 and ≥ 45 years) as well as regional socioeconomic deprivation (Classification into five quintiles, each comprising 20 % of the postal code regions: 1st quintile – low deprivation to 5th quintile – high deprivation). The GDM prevalence was estimated as a 12-month prevalence and corresponds to the proportion of mothers with GDM in relation to all hospital births included in the respective year. In addition, a direct age standardization was applied using the age distribution of the study population from 2021 as the standard population.

## 3. Results and interpretation

**Figure 1:**
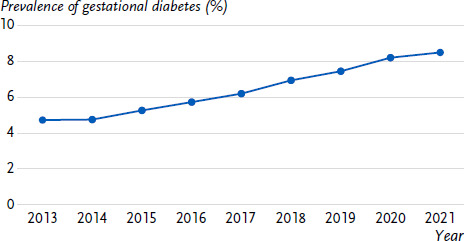
Temporal trend in the age-standardized prevalence of gestational diabetes mellitus from 2013 to 2021; in percent (%) (n per year: Table 1) Source: External inpatient quality assurance for obstetrics at Institute for Quality Assurance and Transparency in Health Care (IQTIG) [13]

After exclusion of women with pregestational diabetes, we included approximately 700,000 hospital birth per year in the analysis (Annex Table 1). From 2013 to 2021, the observed prevalence of GDM rose continuously from 4.6 % to 8.5 % and the age-standardized prevalence from 4.7 % to 8.5 % (Figure 1). In 2021, GDM was documented in 63,563 women. The prevalence of GDM was consistently higher in older compared to younger mothers in all years (Table 1). The increase in prevalence over time was evident in all age groups.

In regions with high socioeconomic deprivation, the age-standardized prevalence of GDM was significantly higher in all years than in regions with low deprivation (Table 1). The prevalence of GDM increased more over time in regions with high socioeconomic deprivation than in regions with low deprivation.

The present results show that the increase in GDM prevalence, which was already visible in an analysis of the years 2013 to 2019 on the same data basis [3], continues in 2020 and 2021. Analyses of outpatient claims data from 2015 to 2020 and statutory health insurance (SHI) data from 2010 to 2020 show a similar increase, with significantly higher prevalence estimates overall [17, 18]. Higher estimates of GDM prevalence in SHI data compared to perinatal statistics data are based on differences in the study population and the case definition of GDM. For example, the perinatal statistics also include women with private health insurance, who differ in their risk profile from women with statutory health insurance. In contrast to a documentation in the maternity record, GDM is defined in the analyses of routine health care data as a single documentation of a GDM diagnosis [19].

When interpreting the development of GDM prevalence over time, several influencing factors must be considered. The increased screening rate since the introduction of screening for GDM in accordance with maternity directive in 2012 [9] is likely to have contributed to a more frequent diagnosis over time. Both an analysis of SHI data (2012: 45.0 %; 2020: 93.3 %) [18] and an analysis based on maternity record data (2016: 83.4 %; 2020: 93.3 %) show that the proportion of pregnant women who receive a test for GDM has increased over time [3, 20].

Also, the frequency of risk factors of GDM has changed over time. The mean maternal age at birth increased from 31.7 to 32.3 [21]. Since the age-standardized and the observed prevalence hardly differ from each other, this could only explain a small part of the increase in prevalence in the present study. Furthermore, the proportion of women with obesity at first antenatal visit during pregnancy rose from 13.6 % in 2013 to 16.8 % in 2021 [22, 23]. While the screening rate in Germany remained unchanged in the first year of the COVID-19 pandemic [20, 24], the German Health Update (GEDA) 2021 study showed an overall increase in body weight and a reduction in physical activity in around a quarter of the adult population compared to the time before the pandemic [25, 26]. Whether this leads to an increase in GDM prevalence analogous to other countries, such as Canada [27], is the subject of further research.

Socioeconomic factors can also influence the risk of GDM [6, 28]. This nationwide analysis shows that the prevalence of GDM is higher in regions with high socioeconomic deprivation than in regions with low deprivation. In addition, these inequalities by regional socioeconomic deprivation have widened over time. The extent to which an increase in the screening rate in socioeconomically deprived regions or the increasing socioeconomic inequality in the risk factors of GDM have contributed to this could not be determined in the present study. An analysis of data from the Bavarian perinatal statistics in combination with a regional deprivation index (Bavarian Index of Multiple Deprivation) revealed this possibility. In 2013 and 2014 (i.e. after the introduction of GDM screening), a higher prevalence of GDM was observed in highly deprived regions than in previous years [29]. The authors concluded that women in highly deprived regions in particular were additionally reached by the screening; it should be noted that the results from Bavaria cannot be generalized to the whole of Germany without further ado. However, socioeconomic inequalities in important risk factors for GDM have also increased over time. For example, analyses of survey data in which socioeconomic status was determined using information on education and income show that the socioeconomic differences in physical inactivity and obesity have increased over time [4, 5]. The same applies to the 5-year risk of type 2 diabetes [30]. However, these analyses assessed the individual socioeconomic status. A link between regional socioeconomic deprivation (measured via GISD) and important common risk factors of GDM and type 2 diabetes [31] as well as the incidence of type 2 diabetes [32, 33] was described in cross-sectional analyses.

## 4. Limitations

The present study is based on all hospital births in Germany. Births taking place outside of the hospital (1 % to 2 % of all births) are not included in the data [34]. The estimation of GDM prevalence is based on the documentation in the maternity record and it is not possible to check whether all cases were documented. However, at least one test for GDM was documented for over 90 % of pregnant women, so we assumed that underreporting is low. The correlation between GDM prevalence and socioeconomic deprivation is based on the linkage of the GISD with the data on GDM at a spatial level. It is therefore not possible to draw conclusions about the connection to the individual socioeconomic status.

## 5. Conclusion

The prevalence of GDM has increased significantly over the period from 2013 to 2021 and is higher in socioeconomically disadvantaged regions than in comparatively wealthier regions. In addition to the introduction of GDM screening in Germany in 2012, the increase in important risk factors for GDM may also have contributed to this increase. As GDM not only affects the health of mother and child around the time of birth, but also entails longer-term health risks, the results underline the need for primary prevention of GDM, i.e. health promotion measures that prevent the occurrence of GDM. These include, for example, promoting exercise, a healthy diet and avoiding obesity before and during pregnancy. As soon as GDM is diagnosed, the quality of medical care with fast and permanent control of maternal blood glucose levels is crucial to prevent health risks for mother and child. When developing and implementing measures, the different living circumstances of women and, in particular, socioeconomically disadvantaged groups should be taken into account. Existing national health targets such as ‘Health around childbirth’ [35] and ‘Type 2 diabetes mellitus’ [36] should be further developed accordingly.

## Key statement

- The age-standardized prevalence of gestational diabetes mellitus in Germany has increased from 4.7 % in 2013 to 8.5 % in 2021.
- In 2021, more than 63,000 women were diagnosed with gestational diabetes mellitus.
- In socioeconomically disadvantaged regions, the prevalence of gestational diabetes mellitus was significantly higher than in regions with low deprivation.

## Figures and Tables

**Table 1:** Temporal trend in the prevalence of gestational diabetes mellitus by age groups and regional socioeconomic deprivation from 2013 to 2021; in percent (%) per year. Source: External inpatient quality assurance for obstetrics at IQTIG [13], GISD Release 2022 v0.2 [14, 15]

Year (N)		2013 (652,315)	2014 (684,610)	2015 (708,135)	2016 (751,771)	2017 (754,219)	2018 (752,463)	2019 (742,116)	2020 (733,467)	2021 (749,690)
**Maternal age at birth in years**										
< 20 years		1.6	1.6	1.7	1.9	2.5	2.6	3.1	3.2	3.6
20 – 24 years		2.8	2.8	2.9	3.4	3.7	4.3	4.7	5.5	5.7
25 – 29 years		3.9	3.8	4.2	4.6	4.9	5.7	6.1	6.7	7.1
30 – 34 years		4.8	4.7	5.2	5.6	6.2	6.8	7.4	8.0	8.3
35 – 39 years		6.0	6.2	6.8	7.5	8.0	8.8	9.4	10.4	10.5
40 – 44 years		7.6	8.2	9.3	9.8	10.5	12.0	12.4	13.8	14.0
≥ 45 years		9.9	11.3	12.2	11.8	14.3	15.8	17.6	17.0	17.2
**Regional socioeconomic deprivation^1, 2^**										
Low	Quintile 1	4.4	4.3	4.5	5.0	5.3	5.9	6.2	6.6	6.8
Medium	Quintile 2	4.9	4.9	5.5	6.1	6.5	7.0	7.1	7.9	8.2
	Quintile 3	4.8	4.6	5.4	5.9	6.4	7.0	7.9	8.7	9.0
	Quintile 4	4.6	4.8	5.6	5.9	6.4	7.4	7.9	8.8	9.1
High	Quintile 5	5.2	5.4	5.8	6.4	7.0	8.0	8.4	9.3	9.7

GISD: German Index of Socioeconomic Deprivation; IQTIG: Institute for Quality Assurance and Transparency in Health Care

^1^Age-standardized prevalence

^2^Due to missing information on place of residence, 3.4 % (n = 219,657) women were excluded from the analysis by regional socioeconomic deprivation

## Appendix

**Annex Table 1:** Study population - number of hospital births stratified by age group and regional socioeconomic deprivation from 2013 to 2021. Source: External inpatient quality assurance for obstetrics at IQTIG [13], GISD Release 2022 v0.2 [14, 15]

Year (N)		2013 (652,315)		2014 (684,610)		2015 (708,135)		2016 (751,771)		2017 (754,219)		2018 (752,463)		2019 (742,116)		2020 (733,467)		2021 (749,690)	
		in %	n	in %	n	in %	n	in %	n	in %	n	in %	n	in %	n	in %	n	in %	n
**Maternal age at birth in years**																			
< 20 years		2.2	14,502	2.2	14,729	2.2	15,225	2.3	17,119	2.0	15,092	1.9	14,056	1.8	13,310	1.7	12,411	1.5	11,033
20 – 24 years		12.2	79,378	11.4	77,944	10.9	77,239	10.8	81,476	10.3	77,916	10.1	76,116	9.9	73,300	9.5	69,449	8.7	65,591
25 – 29 years		28.3	184,333	28.3	193,649	28.5	201,845	28.2	212,012	27.7	209,162	27.1	203,545	26.3	195,482	25.5	187,381	24.7	184,875
30 – 34 years		34.9	227,554	35.3	241,865	35.3	249,766	35.0	262,922	35.6	268,179	36.1	271,452	37.0	274,747	37.9	277,828	38.9	291,632
35 – 39 years		18.3	119,099	18.7	128,085	19.1	135,413	19.6	147,438	20.1	151,904	20.5	154,595	20.6	152,833	20.9	153,041	21.4	160,671
40 – 44 years		4.0	26,070	3.9	26,886	3.8	27,097	3.9	29,104	4.0	30,199	4.1	30,905	4.1	30,689	4.3	31,651	4.6	34,214
≥ 45 years		0.2	1,379	0.2	1,452	0.2	1,550	0.2	1,700	0.2	1,767	0.2	1,794	0.2	1,755	0.2	1,706	0.2	1,674
**Regional socioeconomic deprivation^1^**																			
Low	Quintile 1	24.2	158,119	23.6	161,786	23.0	162,729	22.9	172,165	23.2	175,205	24.5	184,544	23.1	171,062	23.2	169,989	23.5	176,530
Medium	Quintile 2	18.5	120,357	18.5	126,348	18.5	131,254	19.0	142,834	18.5	139,410	18.7	141,025	18.9	140,549	19.0	139,491	19.3	1,346
	Quintile 3	16.5	107,706	16.0	109,376	16.0	113,327	15.5	116,618	16.3	123,250	16.4	123,771	19.2	142,789	19.3	141,820	19.4	145,797
	Quintile 4	20.3	132,532	17.2	118,028	17.1	121,144	20.6	155,132	19.9	150,085	20.6	155,088	17.1	127,141	17.1	125,269	16.9	126,424
High	Quintile 5	18.7	122,213	21.5	147,015	21.2	150,194	18.0	135,020	18.1	136,202	18.1	136,426	17.9	132,587	17.5	128,179	17.1	128,253
Missing		1.7	11,388	3.2	22,057	4.2	29,487	4.0	30,002	4.0	30,067	1.5	11,609	3.8	27,988	3.9	28,719	3.8	28,340

GISD: German Index of Socioeconomic Deprivation; IQTIG: Institute for Quality Assurance and Transparency in Health Care

^1^ Age-standardized prevalence
